# Trade, Diplomacy, and Warfare: The Quest for Elite Rhizobia Inoculant Strains

**DOI:** 10.3389/fmicb.2017.02207

**Published:** 2017-11-09

**Authors:** Alice Checcucci, George C. DiCenzo, Marco Bazzicalupo, Alessio Mengoni

**Affiliations:** Department of Biology, University of Florence, Florence, Italy

**Keywords:** sociomicrobiology, symbiosis, sustainable agriculture, nitrogen-fixation, bacterial genomics

## Abstract

Rhizobia form symbiotic nitrogen-fixing nodules on leguminous plants, which provides an important source of fixed nitrogen input into the soil ecosystem. The improvement of symbiotic nitrogen fixation is one of the main challenges facing agriculture research. Doing so will reduce the usage of chemical nitrogen fertilizer, contributing to the development of sustainable agriculture practices to deal with the increasing global human population. Sociomicrobiological studies of rhizobia have become a model for the study of the evolution of mutualistic interactions. The exploitation of the wide range of social interactions rhizobia establish among themselves, with the soil and root microbiota, and with the host plant, could constitute a great advantage in the development of a new generation of highly effective rhizobia inoculants. Here, we provide a brief overview of the current knowledge on three main aspects of rhizobia interaction: trade of fixed nitrogen with the plant; diplomacy in terms of communication and possible synergistic effects; and warfare, as antagonism and plant control over symbiosis. Then, we propose new areas of investigation and the selection of strains based on the combination of the genetic determinants for the relevant rhizobia symbiotic behavioral phenotypes.

## Rhizobia as a Model for Sociomicrobiologists

Microorganisms have become popular models for addressing sociobiological questions related to the evolution of multicellularity, communication, and to the exchange of nutrients. Indeed, microbial systems are suitable for the study of mutualistic interactions due to their readiness of manipulation, their short generation times, and the simple methods for tracking of resources (West et al., 2006). Moreover, the social behaviors of microorganisms (e.g., cell–cell communication) have profound impacts on human health. In particular, cell–cell communication has been extensively studied with the aim of developing new antimicrobial strategies that block the social organization of pathogenic microorganisms, such as biofilm formation (Xavier, 2016).

Plant-associated bacteria, and more specifically rhizobia, have gained the attention of sociomicrobiologists thanks to the exchange of goods they experience with their host plant (West et al., 2002; Denison et al., 2003; Gubry-Rangin et al., 2010). Rhizobia are a polyphyletic group of *Proteobacteria*, with all rhizobia identified to date belonging to two proteobacterial classes: *Alphaproteobacteria* and *Betaproteobacteria*. Rhizobia live as free bacteria in soil or plants (as commensal endophytes), and may form symbiotic nitrogen fixing associations with compatible and leguminous plants. Fabaceae (Leguminosae) is the third largest family of Angiosperms and includes more than 20,000 species (Lewis, 2005). Access to nitrogen through the symbiosis is an important part of the evolutionary success of legumes, which are able to colonize a multitude of ecosystems and has led to expanded diversity. Moreover, symbiotic nitrogen fixation contributes to legumes being the second most important family of crop plants after the Poaceae; they account for 27% of world grain crop production and contribute up to 33% of the protein in the human diet (Smýkal et al., 2015).

The high economical relevance of the symbiotic interaction has promoted the production of commercial rhizobia inoculants. Inoculation of leguminous crops with rhizobia strains is known to considerably increase crop yield, mediated largely through symbiotic nitrogen fixation (Lupwayi et al., 2006). Soybean in Europe and South America, as well as several legumes in Australia, are probably the most emblematic examples (Thilakarathna and Raizada, 2017). Rhizobia inoculants also influence the overall rhizosphere microbiome, which may lead to biostimulation of other non-legume crops and can inhibit the growth of opportunistic bacterial pathogens (Trabelsi et al., 2011, 2012; Das et al., 2017). Moreover, rhizobia are ubiquitous in soil and in association with plants. Indeed, they can be viewed as plant-associated bacteria that colonize both legumes and non-leguminous plants (Mengoni et al., 2013). Several authors have found rhizobia in the endosphere and rhizosphere of cereals, such as rice [see for instance (Tan et al., 2001; Yanni et al., 2001)] and showed that rhizobia can promote plant growth in these non-legumes species (Bashan et al., 2014; Gopalakrishnan et al., 2015). In sum, rhizobia are important elements of an upcoming new green revolution, where plant-associated microorganisms are key players of eco(nomical) and eco(logical) crop yield increases, both as a direct source of fixed nitrogen in the agroecosystem, as well as through acting as promoters of plant growth and plant health.

During symbiotic interaction, the rhizobia induce the formation of specialized plant structures, known as nodules (Sprent et al., 2017), where nitrogen fixation takes place. Rhizobia provide the plant with fixed nitrogen in the form of ammonia, while the plant provides the rhizobia a homeostatic environment to undergo heterotrophic multiplication using carbon derived from photosynthates and additional micronutrients (e.g., Fe, S, Mo, etc.). Due to this exchange of nutrients, rhizobia have gained attention in sociomicrobiological studies as a model of the evolution of mutualistic interaction. In particular, Hamilton’s rule [for a recent discussion see (Van Veelen et al., 2017)] has been investigated with respect to the exchange of goods taking place between rhizobia and plants (Kiers and Denison, 2008). In fact, by supplying its host with fixed nitrogen, rhizobia strains enhance host growth, thereby potentially increasing its access to photosynthates. However, several rhizobia strains can inhabit nodules found on the same host plant, even co-inhabiting the same nodule (Checcucci et al., 2016). This means that a nitrogen-fixing rhizobia benefits not only its own kin, but also other rhizobia (and non-rhizobia) strains. Those strains compete for host resources and future nodulation opportunities, which paves the way for cheating to occur. However, it has been shown that plants sanction nodules that are inefficient at fixing nitrogen, providing a direct advantage (in the nodule endosphere) only for the mutualistic rhizobia (Kiers et al., 2003).

When considering the molecular mechanisms of social interactions among bacteria, and between plants and bacteria, a plethora of systems can be included, spanning from quorum sensing to volatile molecules, to the classical Nod Factors and flavonoids, to plant immunity cascades and NCR peptides (Mergaert et al., 2006; Van de Velde et al., 2010; Mine et al., 2014; Schulz-Bohm et al., 2015; Kai et al., 2016; Cao et al., 2017). In the last years, studies on competition for nodule occupancy by rhizobia have highlighted the role of several components [for a review see (Onishchuk et al., 2017)]. Here, we divide such components into systems for trade, diplomacy, and warfare. This of course should be considered as an approximation, as in the real global economy, trade and politics have tight and, in some case, combined roles in the same affair.

## Trade

Keeping on the parallelism between mutualistic interaction and biological markets (Werner et al., 2014), the collaborative behavior established by rhizobia and legumes can be compared to a trading partnership. The main good of exchange is fixed nitrogen, passed from bacteroids to the plant cell. In return, the rhizobia are compensated by the plant with space for growth (the nodule) and carbon compounds for the heterotrophic metabolism of the bacteroid. Nodule colonization by rhizobia indeed could be a high remunerative reward offered by the plant. However, the number of rhizobia released back to the soil from dehiscent nodules depends on the type of nodule [indeterminate and determinate, where bacteroids are terminally differentiated or not, respectively (Denison and Kiers, 2004a)], questioning if we can consider legume-rhizobial partnership a full mutualistic interaction (Cao et al., 2017).

Determining the exchange of metabolites (Prell and Poole, 2006; Udvardi and Poole, 2013), and measuring the relative costs invested by the plant and the bacterium during the symbiosis, is necessary to define a predictive model of the metabolic (trade) advantages of symbiotic nitrogen fixation. This model will not be trivial since symbiotic nitrogen fixation is a developmental process. Various cellular metabolites change in relation to the developmental zone of the nodule (White et al., 2007; Ogden et al., 2017). Consequently, the exchange of goods may be related to several biosynthetic and catabolic pathways. Finally, we have to keep in mind that, as large rhizobia genomes provide a high level of metabolic redundancy (diCenzo and Finan, 2015), the (horizontally transferred) dispensable genome fraction may promote considerable strain-specific metabolic variation. Such variability may imply a corresponding variation in the exchange of goods between the plant and rhizobia cells (Remigi et al., 2016). A main point going forward is to better understand this variability in order to select those rhizobia strains more effective in “rewarding” the plant (through the fixed nitrogen offered to the plant) for use as elite inoculants.

## Diplomacy

Aside from the classical exchange of benefits and the reciprocal control of the fitness (trade), the rhizobia–plant mutualism evolved the capacity for partner discrimination. Molecularly, the “entry visa” is based on the lipochitooligosaccharide molecule known as Nod Factor (NF) and NF receptors (Cao et al., 2017). Depending on their particular set of *nod* genes, different rhizobia produce structurally distinguishable NFs that establish the first line of partner choice (Geurts and Bisseling, 2002). However, *nod* genes are not particularly relevant in the selection of elite rhizobia bioinoculants, as they primarily dictate whether a strain can nodulate a specific legume but contribute little to the efficiency of nodulation.

**FIGURE 1:**
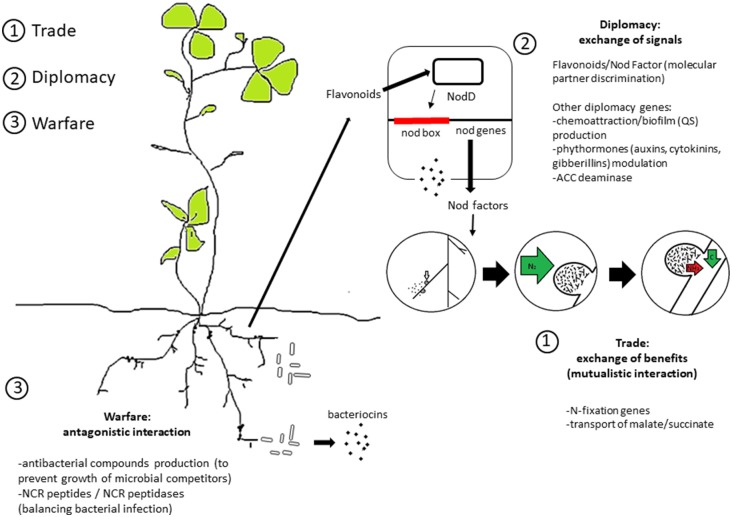
Overview of the genetic and molecular signals of rhizobia social interaction. A schematic division into trade, diplomacy and warfare systems is shown (see text for details).

Instead, the most relevant “diplomacy” genes for selection of elite bioinoculants strains with high competitiveness for nodule occupancy are, in principle, genes related to the modulation of communication and to the direct positive and negative interaction between the plant and bacterium (**Figure 1**). Among the most studied examples is the apparatus involved in quorum sensing. This apparatus is related to phytohormone modulating genes, such as those for auxin production and ACC deaminase that are potentially related to modulation of the rhizobia–legume interaction (Ma et al., 2004; Okazaki et al., 2004; Sugawara et al., 2006; Bianco and Defez, 2009; Checcucci et al., 2017). Additionally, quorum sensing in rhizobia is also linked to the transfer of symbiotic plasmids in several strains, including *Rhizobium leguminosarum* bv. *viciae*, *Sinorhizobium fredii* NGR234, and *Sinorhizobium meliloti* Rm41 [for a review see (Sanchez-Contreras et al., 2007)]. Interestingly, while the quorum sensing apparatus is present in several rhizobia species (González and Marketon, 2003), individual strains may show different responses to quorum sensing signaling molecules (Sanchez-Contreras et al., 2007). This can be related to the large variability in the quorum sensing regulons of rhizobia (Galardini et al., 2015a). It is important to note that symbiotic nitrogen fixation abilities are uncoupled from colonization and nodulation abilities (Westhoek et al., 2017); i.e., rhizobia strains highly competitive for nodule occupancy do not necessarily fix nitrogen efficiently. This has resulted in the evolution of plant imposed sanctions toward ineffective nodules that do not fix nitrogen (Kiers et al., 2003, 2006; Denison and Kiers, 2004b; Kiers and Denison, 2008); otherwise, cheaters (non-mutualist strains) could possibly outcompete the mutualist ones (Gubry-Rangin et al., 2010; Checcucci et al., 2016; Westhoek et al., 2017).

Given that nodulation and nitrogen fixation abilities are not intrinsically linked, it will be necessary to separately consider both processes in the development of elite rhizobia bioinoculants. The exploration of the cornucopia of signaling pathways and of their role in determining the competitive abilities of strains, as well as their effect over the whole rhizosphere microbiome, will probably be one of the greatest challenges of the future. Signaling is mediated at many levels and in many ways, i.e., as cell–cell interaction, diffusible molecules, and possibly by other still poorly known or unknown mechanisms (i.e., volatile organic compounds, electric signals). Moreover, signaling is strongly dependant on the context where strains are growing, and can be influenced by co-resident microbes. Thus signaling cannot be fully examined using conventional microbiological investigations based on pure culture. Reconstructed root microbiotas and easy-to-use gene reporter systems coupled with genome-wide assessments of transcriptional activation during the various stages of symbiosis (Karunakaran et al., 2005; Gao and Teplitski, 2008; Pini et al., 2017) could be a strategy to help define the “diplomatic” relationships of rhizobia strains and the ways to exploit them for elite rhizobia strain selection.

## Warfare

In order for rhizobia to enter into a symbiotic nitrogen fixing relationship with a host plant, they have to effectively compete for rhizosphere colonization with other rhizobia and non-rhizobia. From the bacterial point of view, competition implies preventing the colonization of the niche by other strains. This can take two forms: exploitative (indirect), and interference (direct). Exploitative competition involves more effectively utilizing a common limiting nutrient. As examples, rhizobia can produce siderophores that sequester all available iron for use by themselves, limiting the ability of competitors to grow (diCenzo et al., 2014), or strains can better exploit specific carbon sources present in root exudates (Gage and Long, 1998; Ramachandran et al., 2011). Interference competition, which we refer to as “warfare,” occurs when one cell directly prevents another cell from growing/surviving in the environment. This can occur, for example, through the production of antibacterial compounds, such as bacteriocins, that may play a still poorly defined role in the antagonistic interactions taking place during competition for nodule occupancy (Hirsch, 1979; Oresnik et al., 1999). Bacteriocin-encoding gene clusters can be found in many rhizobial genomes. A search of the IMG database (on August 7, 2017) reported the presence of 11 annotated bacteriocin-related proteins within the members of the genus *Sinorhizobium* (out of 61 genomes, from 1 to 3 per genome), spanning from bacteriocin transporter genes to bacteriocin protection genes. We hypothesize that such bacteriocins may promote antagonistic interactions among members of the same species both in the rhizosphere and within the nodule.

In several host species (e.g., members of the Inverted-Repeat Lacking Clade that includes the majority of agriculturally relevant legumes), plant–rhizobia interaction involves the tight control of bacterial cell proliferation and differentiation based on antimicrobial peptides [nodule cysteine-rich peptides (NCRs)], as well as the suppression of the host immune response mediated through NF (Cao et al., 2017). The evolution of NCR peptides resulted in the emergence of some antagonistic, warfare-like features to the plant–rhizobia interaction. In the classical sense, NCR peptides do not represent warfare, and are perhaps better considered in a “policing” analogy as the “handcuffs” that capture the bacteria within the nodule. Nevertheless, NCR peptides fall into our somewhat broad definition of warfare as they represent a method used by the plant to prevent the bacterium from replicating. NCR peptides promote terminal differentiation of the rhizobia in the nodule, likely increasing the efficiency of nitrogen fixation but preventing the bacterium from ever living as a free-living microbe (increased plant fitness, decreased bacterium fitness). However, on the other hand, rhizobia have evolved NCR peptidases that can cleave NCR peptides, preventing terminal differentiation and nitrogen fixation, but promoting improved bacterial nodule colonization (decreased plant fitness, increased bacterium fitness). Recently, NCR peptidases were found to be involved in determining rhizobia – host compatibility (Price et al., 2015; Wang et al., 2017a,b; Yang et al., 2017). Interestingly, the genes encoding NCR peptidases are harbored by plasmids (Crook et al., 2012), which can be horizontally transmitted and are part of the dispensable genome fraction. This allows for the determination of partner compatibility at the level of a single strain, providing additional complexity to the variability of symbiotic and nitrogen-fixation abilities of possible inoculant strains.

## A Sociomicrobiology and Genomics Assisted Quest for Elite Inoculant Strains

Whereas crop breeding is used to develop new plant cultivars with the desired features, very few research efforts have been dedicated to the development of *ad hoc* rhizobia inoculant strains (Catroux et al., 2001; Sessitsch et al., 2002). There are only a few strains currently used as seed inoculants. They have been selected (when known) on the basis of trials that showed increased crop yield. However, these studies included only a few plant cultivars and few pedo-climatic conditions (Denton et al., 2003). Variables such as plant cultivar, soil pedo-climatic conditions, and more specifically, variables related to the social interaction of rhizobia, have been only cursorily considered.

Elite rhizobia bioinoculants must effectively compete with the native rhizobia for nodule occupancy. This involves appropriately interacting with the soil and rhizosphere microbiomes, and surviving in the soil sufficiently long to interact with the plant. The elite strains should also be highly effective at providing the plant with fixed nitrogen. However, they should also combine a subset of other important, growth-promoting abilities. These could include, for example, producing auxin that can improve legume and non-legume growth, protecting the plant from pathogens, or being able to cope with drought, salt, pollutants, and/or acidic stress conditions. Moreover, but certainly not less important, the elite strain should be amenable to production by industrial fermentation, and stay viable on coated and inoculated seeds as well as in liquid or grain formulations. Finally, the inoculant could have limited year-to-year persistence in unplanted soil, to guarantee the possibility of applying newly improved strains each year without concern of competition from the previous year’s inoculant.

We would argue that taking into account a sociomicrobiology perspective is a necessity to ensure success in the engineering of rhizobia inoculants. This view is re-enforced when considering the effect of the native rhizobia population on the success of rhizobia bio-inoculants. The success of rhizobia inoculants in the cases cited above was partially due to the absence of an established symbiotic rhizobial populations (Deaker et al., 2004). However, for most crops, an indigenous rhizobia can limit, or completely abolish the positive effects of the inoculant [for a review see (Dwivedi et al., 2015)]. This is especially true if the indigenous rhizobia are highly competitive for nodule occupancy but with low nitrogen fixing abilities. Because of this, the competitiveness of rhizobia strains is one of the key aspects that should be considered when inoculant formulations are developed (Lupwayi et al., 2006).

Going forward, attention should be paid to all aspects of the social interactions that rhizobia strains have with the indigenous rhizobia and non-rhizobia rhizosphere population. As reported in the previous sections, social behaviors span from the efficiency of metabolite exchange with the plant, to interactions with the rhizosphere microbiota and access to the nodule endosphere, to the antagonism toward other strains or species, to the modulation of differentiation operated by NCR peptides. Most, if not all, such rhizobia behavioral phenotypes are ideal candidates for the selection of the most appropriate panel of strains to be used as inoculants in agriculture. The knowledge we currently have on the genetic determinants for some of these phenotypes may already permit the *ad hoc* design or selection of improved inoculant strains. Moreover, from an evolutionary point of view, rhizobia present a fascinating panorama to be explored to better understand such social behaviors and the mechanisms that maintain this high diversity of social-related phenotypes, such as selective pressure, drift, and genetic exchange.

The genomes of rhizobia are extremely variable (Maclean et al., 2007), and among species they span from the single large chromosome of ∼9 Mb of the bradyrhizobia, to the highly multipartite genomes of the *Rhizobium* and *Sinorhizobium* genera that include several accessory replicons such as chromids, megaplasmids, and smaller plasmids (Galibert et al., 2001; Tian et al., 2012). Rhizobia also have large pangenomes, with a considerable portion of their genome consisting of accessory and dispensable genes (Tian et al., 2012; Galardini et al., 2015b; Young, 2016). For rhizobia with multipartite genomes, a functional modularity of the genome has been shown (Galardini et al., 2013, 2015a; diCenzo et al., 2016). Additionally, the secondary replicons are generally more genetically diverse between strains than the primary chromosome (Galardini et al., 2013). These properties can, in theory, permit the construction of “hybrid” strains by combining accessory replicons that harbor gene functions related to competition, symbiotic efficiency, and possibly tolerance to harsh soil conditions, from different donor strains in a single genome. This would be similar to the approach recently employed by Agnoli et al. (2017) with *Burkholderia cepacia* complex species. Additionally, the large pangenomes may harbor many genes related to the important “social” phenotypes referred in the previous paragraphs. Such “social” genes may be useful candidates for strain improvement and may be combined together (in a single strain) by shuffling secondary replicons from different donors. We can imagine the development of an approach to promote horizontal gene transfer of such genetic determinants between strains in a sort of “pangenome-assisted selection,” conceptually analogous to the marker-assisted selection in crops (Collard and Mackill, 2008). In this process, we could choose (select) from the pangenomic space of a given rhizobial species those genes that can, once combined in a single strain, provide the elite features. However, to efficiently deal with “pangenome-assisted selection,” new experimental settings and knowledge are compulsory to (i) perform high throughput screenings of the symbiotic potential of rhizobial strains, and (ii) have detailed information about the signaling between rhizobia, the plant, and the rhizosphere microbiota. Experiments aimed at deciphering the exchange of signals and the effect on plant growth in reconstructed root microbiotas are needed to move toward a more comprehensive view of the intimate sociobiological relationship between the plant and its rhizobial partners, and then promote a rationale selection of elite rhizobial inoculants.

## Author Contributions

AM conceived the study. AC drafted the manuscript. GD and MB contributed discussing the ideas and contributed in drafting the manuscript. All authors read and approved the final version of the manuscript.

## Conflict of Interest Statement

The authors declare that the research was conducted in the absence of any commercial or financial relationships that could be construed as a potential conflict of interest.
